# Prevalence and Predictors of Functional Vitamin K Insufficiency in Mothers and Newborns in Uganda

**DOI:** 10.3390/nu7105408

**Published:** 2015-10-16

**Authors:** Data Santorino, Mark J. Siedner, Juliet Mwanga-Amumpaire, Martin J. Shearer, Dominic J. Harrington, Unni Wariyar

**Affiliations:** 1 Department of Pediatrics and Child Health, Mbarara University of Science and Technology, Plot 8-18 Mbarara–Kabale Road, P.O. Box 1410, Mbarara, Uganda; jmwanga@must.ac.ug (J.M.-A.); wariyarunniwariyar@gmail.com (U.W.); 2 Department of Medicine and Infectious Diseases, Massachusetts General Hospital and Harvard Medical School, 125 Nashua Street, Boston, MA 02114, USA; MSIEDNER@mgh.harvard.edu; 3 Centre for Haemostasis and Thrombosis, St. Thomas’ Hospital, Westminster Bridge Road, London SE1 7EH, UK; Martin.Shearer@gstt.nhs.uk (M.J.S.); Dominic.Harrington@viapath.co.uk (D.J.H.)

**Keywords:** vitamin K, undercarboxylated prothrombin, deficiency, insufficiency, newborn, bleeding, haemorrhage, prophylaxis

## Abstract

Vitamin K deficiency bleeding (VKDB) in infancy is a serious but preventable cause of mortality or permanent disability. Lack of epidemiologic data for VKDB in sub-Saharan Africa hinders development and implementation of effective prevention strategies. We used convenience sampling to consecutively enroll mothers delivering in a southwestern Uganda Hospital. We collected socio-demographic and dietary information, and paired samples of maternal venous and neonatal cord blood for the immunoassay of undercarboxylated prothrombin (PIVKA-II), a sensitive marker of functional vitamin K (VK) insufficiency. We used univariable and multivariable logistic regression models to identify predictors of VK insufficiency. We detected PIVKA-II of ≥0.2 AU (Arbitrary Units per mL)/mL (indicative of VK insufficiency) in 33.3% (47/141) of mothers and 66% (93/141) of newborns. Importantly, 22% of babies had PIVKA-II concentrations ≥5.0 AU/mL, likely to be associated with abnormal coagulation indices. We found no significant predictors of newborn VK insufficiency, including infant weight (AOR (adjusted odds ratio) 1.85, 95% CI (confidence interval) 0.15–22.49), gender (AOR 0.54, 95% CI 0.26–1.11), term birth (AOR 0.72, 95% CI 0.20–2.62), maternal VK-rich diet (AOR 1.13, 95% CI 0.55–2.35) or maternal VK insufficiency (AOR 0.99, 95% CI 0.47–2.10). VK insufficiency is common among mothers and newborn babies in southwestern Uganda, which in one fifth of babies nears overt deficiency. Lack of identifiable predictors of newborn VK insufficiency support strategies for universal VK prophylaxis to newborns to prevent VKDB.

## 1. Introduction

Vitamin K Deficiency Bleeding (VKDB) in infancy is a rare but potentially serious worldwide problem with a high risk of mortality or permanent disability, primarily due to the high incidence of intracranial haemorrhage (ICH) of the later onset syndrome [1,2,3,4,5]. It is a disease of breastfeeding infants [1,6,7,8,9] and can be prevented by administration of vitamin K (VK) to newborns shortly after birth [1,9,10,11,12]. VKDB is classified according to the age of presentation as early, classical and late [1]. Early VKDB occurs in the first 24 h after birth, it is generally rare and often associated with maternal anticoagulant and or anticonvulsant usage during pregnancy. Classical and late VKDB occur between days 2 to 7, and days 8 to 6 months of life, respectively [9]. In classical VKDB, bleeding typically occurs from the gastrointestinal tract, umbilicus, skin, nose or after circumcision [1,9,10] while in late VKDB, bleeding predominantly occurs within the brain with prevalence rates of ICH as high as 60%–80% [2,3]. Reported incidence rates of classical and late VKDB over the last 50 years in infants who have not been given VK prophylaxis vary widely across different countries.

Apart from reflecting the lack of standardization for case definitions [4,10], there is evidence that the incidence of VKDB reflects economic and nutritional status [6,8,9], social customs such as male circumcision [6,13], and probably genetic variations such as those known to influence the metabolism and intracellular recycling of VK [14]. For classical VKDB, the extremely high incidence rates of 1.7% in Cincinnati, USA in the 1960s [6] and 0.8% in Addis Ababa, Ethiopia from 1982–1991 [8] are noteworthy for the economic deprivation of the populations studied. Incidence rates were strongly influenced by male circumcision in the Cincinnati study [6] but not in the Addis Ababa study where circumcision occurred after 7 days of age and outside the window of classical VKDB [8]. In contrast, the incidence of classical and late VKDB from national surveys in two European countries (Germany and the British Isles) in the late 1980s reveal a markedly lower incidence of 4–7 cases per 10^5^ births in infants given no VK prophylaxis [9,10,15]. A much higher prevalence of late VKDB has been reported in countries of South East Asia (e.g., Japan, Thailand, Malaysia, Vietnam and China) with incidence rates ranging from 11 to 116 cases per 10^5^ births [3,4,9,16]. There is no data for the prevalence of late VKDB in sub-Saharan Africa. Apart from exclusive breastfeeding [1,6,7,8,9], other known risk factors for classical and late VKDB are diarrhea, prolonged antibiotic use and the presence of underlying diseases (e.g., biliary atresia, alpha-1-antitrypsin deficiency, cystic fibrosis) causing malabsorption of VK [1,5,17,18]. Feeding infants with milk formulas (both unsupplemented and supplemented) offers a high protection against VKDB owing to their higher VK content compared to breast milk [19,20] resulting in a greatly superior VK status of formula-fed infants [21].

The lack of data about the epidemiology of VKDB in sub-Saharan Africa has limited the development and implementation of guidelines for prevention of VKDB. As such, VK is not typically listed as a priority drug for children by programmatic organizations such as in the World Health Organization’s Model Medicines list for children [13]. To estimate the incidence of neonatal haemorrhage in Uganda, we performed a retrospective chart review from June–August 2010 at Mbarara Regional Referral Hospital (MRRH) in Southwestern Uganda where VK prophylaxis is not routinely administered to newborns at birth. We found a 6.6% incidence of haemorrhage among all neonatal admissions to the Hospital during the observation period (unpublished data). This was higher than the neonatal haemorrhage prevalence previously reported by Lulseged in Ethiopia [8]. Based on this high prevalence of newborn haemorrhage, we conducted a study to determine the epidemiology and predictors of functional VK insufficiency among mothers and their newborns at MRRH. We define “functional VK insufficiency” as a nutritional state in which the hepatic stores of vitamin K are insufficient to ensure full gamma-glutamyl carboxylation of the VK-dependent procoagulant proteins, factors II (prothrombin), VII, IX and X. In states of VK insufficiency, functionally defective undercarboxylated species of factors such as prothrombin are released into the bloodstream where they can be measured [9,22]. Historically the collective name for these undercarboxylated species is PIVKA (Proteins Induced by vitamin K Absence or Antagonism). In the present study functional VK insufficiency was assessed by the measurement of undercarboxylated prothrombin (PIVKA-II) which has been shown to be a highly sensitive and selective functional marker of VK status with respect to its coagulation function [9,22]. Our choice of PIVKA-II has several advantages over traditional biomarkers of VK status. First, global coagulation tests such as the prothrombin time (PT) or activated partial thromboplastin time (APTT) are insufficiently sensitive to detect early VK insufficiency. As reviewed by Suttie [23], a 1–2 second increase in the PT only occurs when the percent of active (carboxylated) prothrombin has dropped to below 50% of normal whereas immunoassays of PIVKA-II can readily detect undercarboxylated species of prothrombin when there are no observable changes in the PT. For example, even when the concentration of active prothrombin is as high as 90% there is a 20-fold increase in the concentrations of PIVKA-II as measured by an immunoassay such as that used in our study [23]. Second, a prolonged PT lacks specificity for VK insufficiency: for example in a VK supplementation trial carried out in 98 preterm infants, 21 infants who had a significantly prolonged PT were shown to have satisfactory VK status as assessed by serum PIVKA-II and phylloquinone measurements [24]. Thus while a prolonged PT (and APTT) are expected findings in advanced vitamin K deficiency, they have important limitations as early biomarkers of VK insufficiency.

## 2. Experimental Section

### 2.1. Enrollment and Study Procedures

We consecutively enrolled mothers delivering during daytime hours on the peripartum ward at MRRH. Women in active labour were consented before the onset of second stage of labor and were followed until delivery for blood collection at birth. Women with self-reported liver disease, visible jaundice or taking warfarin were excluded [25,26]. We used interviewer administered structured questionnaires to collect socio-demographic data. We also recorded gestational age, newborn weight, and newborn gender. We assessed maternal diet using a 22-item non-validated, and non-quantitative food frequency questionnaire to determine intake frequency of VK rich food [27]. Participants stated daily, weekly and monthly frequency with which they ate VK-rich foods using an interviewer administered food frequency questionnaire. We defined a frequent VK rich food intake as five or more servings per week of green vegetables and or peas.

### 2.2. Blood Collection and Laboratory Procedures

Maternal venous blood from the brachial vein and newborn cord blood were collected immediately at birth in the delivery room. Serum was separated and stored at negative 70 degrees Celsius. At the conclusion of study procedures, samples were transported by courier on dry ice to the Centre for Haemostasis and Thrombosis, Guy’s and St. Thomas’ Hospital Foundation Trust in London for analysis. We measured undercarboxylated prothrombin (factor II) also known as Protein Induced by vitamin K Absence-II (PIVKA-II) serum concentrations using a conformation-specific PIVKA-II monoclonal antibody with a sandwich ELISA [22,28,29,30]. Concentrations of PIVKA-II were expressed as Arbitrary Units per mL (AU/mL) with 1 AU/mL being equivalent to 1 µg of multiple species of uncarboxylated or partially carboxylated prothrombin purified by electrophoresis [22,28,29,30]. Using this same immunoassay, PIVKA-II concentrations in healthy VK replete adults and infants are <0.20 AU/mL. Newborn babies with PIVKA-II values ≥0.20 AU/mL are defined as having vitamin K insufficiency with values ≥5.0 AU/mL approaching a state of overt VK deficiency that are likely to be associated with abnormal coagulation indices and which are clinically relevant to bleeding risk [22]. However, it was not possible to perform any coagulation tests (e.g., prothrombin time) in our study.

### 2.3. Statistical Analyses

Our primary outcome was VK insufficiency at birth as defined by PIVKA-II of greater than or equal to 0.2 AU/mL. Exposures of interest were newborn weight, gender, gestational age [31] and maternal intake of VK rich foods [25]. We fitted univariable and multivariable logistic regression models to estimate associations between the primary outcome of interest (raised PIVKA-II) and exposures of interest. All variables for which we had a priori reason to consider as potential confounders or correlates of vitamin K insufficiency were maintained in the multivariable model. A *p*-value of less than or equal to 0.05 was considered statistically significant [32]. Data analysis was performed with Stata Version 12 (Statcorp, College Station, TX, USA).

### 2.4. Ethics Statement

All participants gave signed informed consent. All babies received intramuscular prophylactic VK after blood sample collection. The study procedures were reviewed and approved by Mbarara University Faculty of Medicine Research and Ethics committee, the Institutional Review Committee at the Mbarara University of Science and Technology and the Uganda National Council for Science and Technology.

## 3. Results

We enrolled 141 mother-baby pairs over a period of 4 months (June–September). The median maternal age was 25 years (Interquartile range: 21–29 years). All mothers approached to participate in the study, gave their written consent. All samples analyzed yielded useable laboratory values. Sixty six percent (93/141) of mothers came from town/urban settings mainly from Mbarara town where MRRH is located. Ninety six (136/141) of the women were married, 57.5% had no secondary education, and 33.3% were primigravidae. Sixty four percent of babies (90/141) were male, 9% (13/141) were preterm (born before 37 completed weeks of amenorrhea) and 3% (4/141) had low birth weight (<2.5 kg). The median birth weight was 3.2 kg (IQR: 2.9–3.8 kg). Spontaneous vaginal delivery accounted for 75% (107/141) of deliveries, caesarian section, 23.4% (33/141) and only one baby was delivered by vacuum extraction. VK insufficiency defined by a detectable PIVKA-II of ≥0.2 AU/mL was present in 33% (47/141) of mothers and 66% (93/141) of babies as shown in Table 1. Six sets of twins were enrolled in the study, there was no PIVKA-II value-category difference between twin siblings, therefore, for the purposes of this study, the PIVKA-II value of the first-born twin were used for analysis. Highly elevated, concentrations of PIVKA-II (≥5.0 AU/mL) indicative of likely abnormal coagulation [22] were found in 22% (31/141) of newborns but in none of their mothers. No newborn babies had detectable postpartum bleeding before hospital discharge. However, all newborn babies received intramuscular vitamin K within one hour of delivery during the period of the study irrespective of study enrolment status.

**Table 1:** Maternal dietary habits and newborn characteristics at birth for the total cohort and by presence of vitamin K (VK) insufficiency.

Variable	Total Cohort (*n* = 282)	PIVKA-II ≥ 0.2 AU/mL ^έ^ (*n* = 140)	PIVKA-II < 0.2 AU/mL ^π^ (*n* = 142)	*p*-Value
Mothers, % (*n*)	100 (141)	33 (47)	67 (94)	
Newborn babies, % (*n*)	100 (141)	66 (93)	34 (48)	
Female newborn, % (*n*)	36 (51)	57 (29)	43 (22)	0.086
Low birth weight (<2.5 kg), % (*n*)	3 (4)	75 (3)	25 (1)	0.699
Preterm, % (*n*)	9 (13)	62 (8)	38 (5)	0.724
Maternal VK-rich intake *, % (*n*)	40 (56)	32 (18)	68 (38)	0.808

* Vitamin K-rich intake as defined by intake of greens and or peas five or more times a week; ^έ^ PIVKA-II: Protein induced in vitamin K absence, with a level ≥ 0.2 Arbitrary Units indicative of vitamin K insufficiency; ^π^ PIVKA-II level < 0.20 AU/mL indicative of normal vitamin K status. AU/mL: Arbitrary Units per mL.

We found no significant predictors of newborn VK insufficiency among newborn or maternal characteristics including: infant weight (AOR (adjusted odds ratio) 1.85, 95% CI (confidence interval) 0.15–22.49), gender (AOR 0.54, 95% CI 0.26–1.11), term birth (AOR 0.72, 95% CI 0.20–2.62), maternal VK-rich diet (AOR 1.13, 95% CI 0.55–2.35) and maternal VK insufficiency (AOR 0.99, 95% CI 0.47–2.10) as shown in Table 2.

**Table 2:** Univariate and multivariate logistic regression models of correlates of vitamin K (VK) insufficiency in newborns, as defined by a PIVKA II concentration ≥ 0.2 AU/mL.

	Univariable Logistic Regression		Multivariable Logistic Regression	
Characteristic	AOR (95% CI)	*p*-Value	AOR (95% CI)	*p*-Value
Female newborn	0.54 (0.26–1.09)	0.088	0.54 (0.26–1.11)	0.093
Low birth weight (<2,500 g)	1.57 (0.16–15.48)	0.701	1.85 (0.15–22.49)	0.630
Preterm (<37 weeks of gestation)	0.81 (0.25–2.62)	0.725	0.72 (0.20–2.62)	0.619
Maternal VK insufficiency	1.00 (0.48–2.09)	1.000	0.99 (0.47–2.10)	0.983
Maternal VK-rich intake *	0.915 (045–1.88)	0.808	1.13 (0.55–2.35)	0.737

* Vitamin K rich intake as defined by intake of greens and or peas five or more times a week; PIVKA-II: Protein induced in vitamin K absence, with a level ≥ 0.2 Arbitrary Units indicative of vitamin K insufficiency. AOR: adjusted odds ratio; CI: confidence interval.

Up to 61.5% (8/13) of all premature babies had detectable PIVKA-II and 23% (3/13) had highly elevated PIVKA-II values (≥5 AU/mL).

## 4. Discussion

Among mothers delivering at a publicly-operated referral hospital in rural southwestern Uganda, VK insufficiency is very common in both mothers (33%) and their newborns (66%). Our study is the first to characterize the prevalence of VK insufficiency in a Sub-Saharan Africa setting using the gold standard measurement (PIVKA-II assay) for functional VK insufficiency with respect to its coagulation function. We found no maternal or infant clinical or dietary predictors of VK insufficiency, implying that routine, prophylactic VK administration should continue to be the standard of care for prevention of VKDB in newborns. Given the high prevalence of VK insufficiency and potential devastating effects of VKDB, availability of VK should be prioritized in resource-limited settings, and especially in health facilities that perform perinatal care.

The 33% prevalence of functional VK insufficiency among mothers in our study is notable and higher than other previous reports. For example, using the same PIVKA-II assay, Chuansumrit *et al.*, found a prevalence of VK insufficiency of 12% among mothers delivering at Ramathibodi hospital in Thailand [22], a country in which the incidence of VKDB was estimated to be 72 cases per 10^5^ births before universal prophylaxis was introduced [3]. Lower dietary intakes of VK by Ugandan mothers compared to Thai mothers may explain the high prevalence of VK insufficiency in our study population. However, the two studies used different methods to measure VK intake, limiting direct comparability.

Similarly, the prevalence of detectable PIVKA-II of 66% in newborn babies in our study was much higher than the 16% prevalence in the Thai study [22] or the 23% prevalence in a study of preterm infants in England [29], all done with the same PIVKA-II assay. Another notable difference between this Ugandan and the Thai study was that 22.0% (31/141) of Ugandan babies had clinically significant PIVKA-II concentrations (≥5.0 AU/mL) compared to 1.5% (10/683) of Thai babies despite the fact that both cohorts were comparable in their birth characteristics. PIVKA-II concentrations greater than 5 AU/mL are likely associated with abnormal coagulopathy and an increased risk for spontaneous bleeding [22].

We found no consistent correlation between the presence or absence of detectable PIVKA-II in cord blood with its presence or absence in the respective paired sample from the mother. In other words, the concentration of PIVKA-II in the mother was not predictive of the concentration in her newborn baby. We should emphasize however that while highly raised PIVKA-II concentrations (≥5.0 AU/mL) were found in 22% of newborns, none of their mothers showed evidence of such a similarly severe degree of VK insufficiency. In addition, the frequency of intakes of VK-rich foods in mothers was not predictive of the functional VK status of their newborns. This lack of correlation between maternal and newborn vitamin K status has been reported previously in a much smaller European study of PIVKA-II measurements in 22 infant-mother pairs [33], but the reasons for this disparity remain unexplained. Certainly, case reports have linked severe maternal dietary VK deficiency to intracranial bleeding in fetal life showing the importance of maintaining adequate maternal intakes of vitamin K during pregnancy [34,35]. Importantly, the lack of clear predictors or correlates of VKD in women or their babies reinforce the need for routine VK administration to newborn babies independent of maternal or newborn factors to help prevent VKDB.

We acknowledge limitations to our study, most importantly the use of a non-standardized dietary questionnaire for determination of VK intake. It should also be noted that our sample size and confidence intervals did not exclude the possibility of a type II error. For example, our estimated adjusted odds of VK insufficiency for each kilogram of birth weight was 1.85 (95% CI 0.15–22.49), suggesting that a significant effect of birth weight on VK insufficiency might have been detected with a larger sample size. To address these shortcomings and further explore these relationships, we intend to conduct a multicenter clinical study to measure VK intakes using a more accurate dietary assessment method (e.g., weighed dietary records) and the prevalence of VK insufficiency in pregnant women and their newborn babies in Sub-Saharan Africa. This additional data will broaden our understanding of the epidemiology of VK intake and deficiency, and help guide recommendations for VK requirements and administration in the region. The study also is characterized by a convenience sampling method in a regional referral Hospital located in an urban area in southwestern Uganda, which might affect generalizability to mothers in rural areas or those without means to reach a referral hospital.

## 5. Conclusions

Functional VK insufficiency evidenced by raised PIVKA-II is common among mothers and newborn babies in southwestern Uganda. Worryingly, 22% of babies had highly elevated PIVKA-II concentrations that are likely to be associated with an increased risk of bleeding. We found no predictors of newborn VK insufficiency, supporting a strategy of universal administration of newborn vitamin K prophylaxis to prevent VKDB.
